# Cytoprotective potency of naringin against di-n-butylphthalate (DBP)-induced oxidative testicular damage in male rats

**DOI:** 10.1007/s00210-023-02874-y

**Published:** 2023-12-13

**Authors:** Anis Anis, Sameh H. El-Nady, Hany A. Amer, Hamed T. Elbaz, Ahmed E. Elweza, Nermeen Borai El-Borai, Salah S. El-Ballal

**Affiliations:** 1 Department of Pathology, Faculty of Veterinary Medicine, University of Sadat City, Sadat City, 32897 Egypt; 2 Department of Pathology, Animal Reproductive Research Institute, Giza, 12556 Egypt; 3 Department of Theriogenology, Faculty of Veterinary Medicine, University of Sadat City, Sadat City, 32897 Egypt; 4 Department of Forensic Medicine and Toxicology, Faculty of Veterinary Medicine, University of Sadat City, Sadat City, 32897 Egypt

**Keywords:** Naringin, Di-n-butyl phthalate, Oxidative stress, Spermatozoa, Testosterone, Testicular degeneration

## Abstract

The present study aimed to investigate the protective potential of naringin (NG) against di-n-butyl phthalate (DBP)- induced testicular damage and impairment of spermatogenesis in rats. Forty-two male Wistar albino rats were divided into six equal groups, and treated orally, 3 times weekly for 8 successive weeks. Control vehicle group was administrated olive oil, naringin-treated group was administered NG (80 mg/kg), DBP 250- and DBP 500- intoxicated groups received DBP (250 mg/kg) and (500 mg/kg), respectively, NG + DBP 250 and NG + DBP 500 groups received NG, an hour prior to DBP 250 and 500 administration. The results revealed that DBP induced dose-dependent male reproductive dysfunctions, included a significant decrease in the serum testosterone level concomitantly with significant decreases in the sperm count, viability, and total motility. Meanwhile, DBP significantly increased the testicular malondialdehyde level with significant reductions of glutathione content and catalase activity. Histopathologically, DBP provoked absence of spermatozoa, degenerative changes in the cell layers of seminiferous tubules and a significant decrease in the thickness of the seminiferous tubules epithelium. Conversely, the concomitant treatment with NG, one hour before DBP 250 or 500- intoxication mitigated the dose-dependent reproductive dysfunctions induced by DBP, evidenced by significant increases of serum testosterone level, sperm motility, count and viability along with marked improvement of the oxidant/antioxidant status and testicular histoarchitecture. In conclusion, the findings recorded herein proved that NG could mitigate DBP-induced testicular damage and impairment of spermatogenesis, suggesting the perspective of using NG as a natural protective and therapeutic agent for alleviating the reproductive dysfunctions and improving reproductive performance, mainly via its potent antioxidant activity.

## Introduction

Over the last decades, considerable attention has been raised about the male reproductive disorders and the frequent increase in the rate of male infertility developed over a short period of time, suggesting the implication of various environmental factors and contaminants (Krzastek et al. 2020) (. Phthalates are ubiquitous environmental contaminants identified as endocrine disruptors. Di-n-butyl phthalate (DBP), the most common phthalate, is predominantly used in the manufacture of many industrial products, including polyvinyl chloride plastics, printing inks, food packaging products, medical devices, insecticides, and some cosmetic and pharmaceutical formulations (Zeng et al. 2013), resulting in the indiscriminate release of DBP to the environment and providing wide-spread animal and human exposure (Guerra et al. 2010). Generally, human exposure occurs primarily via food and water preserved in plastic or other packing materials containing DBP. Pharmaceutical formulations also result in human oral exposure, because DBP is used to coat medicines such as antibiotics, antihistamines, and laxatives (Schettler 2006; Zhou et al. 2010). Additionally, humans are directly exposed to DBP through dermal contact with DBP- containing products or inhalation of contaminated air (Wittassek et al. 2011). Recently, many studies suspected the involvement of DBP and other phthalates in male reproductive health problems, mainly due to their endocrine-disrupting effects (Zhou et al. 2010; 2011). Several mechanisms have been implicated in the DBP- induced reproductive disorders, including deterioration of sperm quality (Aly et al. 2016), male reproductive tract deformities (Liu et al. 2016), Sertoli and Leydig cell dysfunctions (Bielanowicz et al. 2016), impairment of male fertility, spermatogenesis and steroidogenesis (Nelli and Pamanji 2017), and apoptosis of spermatogenic cells (Alam and Hoque 2018). Oxidative stress and lipid peroxidation are the main possible mechanisms by which DBP could induce male reproductive disorders as a result of high polyunsaturated fat content in the membrane of sperm **(**Zhou et al. 2010, 2011; Nelli and Pamanji 2017). Accordingly, the use of natural antioxidants may play a pivotal role in enhancing reproductive performance and alleviating the reproductive disorders induced by a variety of environmental contaminants. Citrus peel, a citrus by-product, is a rich source of bioflavonoids which are considered potent bioactive agents against oxidative stress and lipid peroxidation (Koolaji et al. 2020). Naringin is a dietary bioflavonoid richly found in citrus fruit peel and hydrolyzed by intestinal microflora, yielding a highly absorbed form, naringenin (Chen et al. 2014). Both naringin and its metabolite naringenin, proved diverse biological and pharmacological activities, including antimicrobial, anti-atherogenic, antioxidant, anti-inflammatory, and anti-carcinogenic activities (Chen et al. 2016; Salehi et al. 2019). Additionally, NG exhibits therapeutic potential for the treatment of multiple illnesses, including diabetes (Punithavathi et al. 2008), cardiovascular and metabolic disorders (Alam et al. 2013, 2014), hepatic, renal and testicular damages (Adil et al. 2014, 2015), neurodegenerative disorders (Gopinath and Sudhandiran 2016) and respiratory diseases (Shi et al. 2017). In the light of this background, this study aimed to investigate the possible protective effect of NG against DBP-induced testicular damage and impairment of spermatogenesis in rats.

## Materials and methods

### Materials

Di-n-butyl phthalate (DBP), Dibutyl benzene-1, 2-dicarboxylate, (CAS No.84–74-2; purity of 99%) was purchased from Sigma–Aldrich Company. Naringin (NG) from citrus fruit (flavanone-7-O-glycoside) (CAS No. 10236–47-2; purity of ≥ 90%) was purchased from Sigma–Aldrich Company. Diagnostic Enzyme-Linked Immunosorbent Assay (ELISA) kit for assessment of serum testosterone level was purchased from DiaSorin Company, and kits for assessment of testicular content of malondialdehyde (MDA), reduced glutathione (GSH) and catalase (CAT) were purchased from Biodiagnostic Company Dokki, Giza, Egypt. Other chemicals for sperm evaluation and histopathological examination were of analytical grades and commercially available.

### Animals and experimental design

Forty-two healthy adult male Wistar albino rats (3 months old and of weight rang 180–200 g) were purchased from Alzyade Experimental Animals Production Center, Giza, Egypt. Rats were housed in polypropylene cages with mesh wire tops and kept at natural ventilated room under a standard laboratory condition (28 ± 2 °C, 50–65% relative humidity and natural daily dark/light cycle), provided with a standard commercial diet and clean tap water ad libitum water during the acclimatization period and throughout the experiment. The animals care and handling followed the ethical guidelines of the International Animal Care and Use Committee IACUC, Faculty of Veterinary Medicine, University of Sadat City (Approval No. VUSC-014–1-19).

Rats were randomly divided into six groups (*n* = 7), and treated orally by gavage, 3 times a week for 8 successive weeks.

- *Control vehicle group:* Rats received olive oil (vehicle of DBP).
- *Naringin (NG) group:* Rats were administered NG (80 mg/kg/day) dissolved in distilled water (Arumugam et al. 2016).
- *Di-n-butylphthalat 250 (DBP 250) group:* Rats were administered DBP (250 mg/kg/day) dissolved in olive oil (Yin et al. 2016).
- *Di-n-butylphthalat 500 (DBP 500) group:* Rats were administered DBP (500 mg/kg/day) dissolved in olive oil (Yin et al. 2016).
- *Naringin and Di-n-butylphthalat 250 (NG* + *DBP 250) group:* Rats were administered NG (80 mg/kg/day), an hour before DBP (250 mg/kg/day) intoxication.
- *Naringin and Di-n-butylphthalat 500 (NG* + *DBP 500) group:* Rats were administered NG (80 mg/kg/day), an hour before DBP (500 mg/kg/day) intoxication.

### Sample collections

By the end of the experiment, rats were fasted overnight, anesthetized by inhalation of isoflurane and prepared for samples collection. Blood samples were collected from the retro-orbital plexus and centrifuged at 3000 rpm at 4°C for 15 min and the collected sera samples were kept at -20°C for hormonal assay. After animals were euthanized by cervical dislocation, testes were collected and the caudal epididymis of each testis was immediately excised, cleared of adhering tissues, dissected out gently to release the sperms into 1 ml of pre-warmed phosphate buffer saline, and then incubated at 37°C for 20 min for further sperm assessment (Talebi et al. 2007). One of the excised testes was perfused with physiological saline and stored at -80°C for further tissue antioxidant analysis, while the other one was fixed in neutral-buffered formalin 10% for histopathological investigation.

### Serum hormonal assay

Serum testosterone level was assessed according to Feldman et al (2002), using diagnostic ELISA kit and following the manufacturer's protocol.

### Evaluation of sperm quality

Sperm quality was estimated by evaluating sperm count, motility and viability according to the methods described by Mousa et al (2020). Total sperm motility (%) was evaluated by adding 20 μl of sperm suspension on a warm glass slide and covered with a warm coverslip and estimated using phase-contrast microscope (X40) with a hot stage (37°C), and then classified as a proportion of progressive (rapid and slow) and non-progressive. Similarly, the immotile sperm (%) was also recorded. Sperm count was determined by allocating 10 µl of sperm suspension in Neubauer chamber. Sperm viability was evaluated by mixing 10 µl of sperm sample with 10 µl eosin stain (0.5%) on a microscope slide and covered with a coverslip and then a total of 200 sperm were counted and specified as live (unstained) and dead (red stained) sperm using light microscopy (X40).

### Assessment of oxidant/antioxidant biomarkers

The preserved testis of each rat was homogenized in cold PBS (pH 7.4), centrifuged at 4000 rpm at 4°C for 15 min, and the obtained supernatants were used for investigation of MDA level, GSH content, and CAT activity, following the manufacturer's instructions of the commercial kits and according to the methods described by Ohkawa et al (1979), Beutler et al. (1963) and Aebi (1984), respectively.

### Histopathological Examination

The formalin- fixed testes were routinely processed, embedded in paraffin wax, sectioned with a microtome (3–5 μm thicknesses), stained with hematoxylin and eosin (H&E) stain according to Bancroft and Layton (2013), and photographed by using Lieca DMLB microscopes and Leica EC3 digital camera. Additionally, the thickness of seminiferous tubules epithelium (um) were assessed using computer software-assisted image analysis (Image J, version 1.4.3.67; National Institutes of Health, Bethesda, Maryland, USA) (Tracey et al. 2014), and the comparative histopathological differences between the different treated groups were analyzed by SPSS.

### Statistical analysis

The obtained data were subjected to ANOVA followed by Duncan's Multiple Range test for post hoc analysis using SPSS software, version 16 (released in 2007) and are presented as means ± S.E. Statistical differences were set at *P* < 0.05.

## Results

### Naringin increased the di-n-butyl phthalate- induced decrease in serum testosterone level

**Fig. 1:**
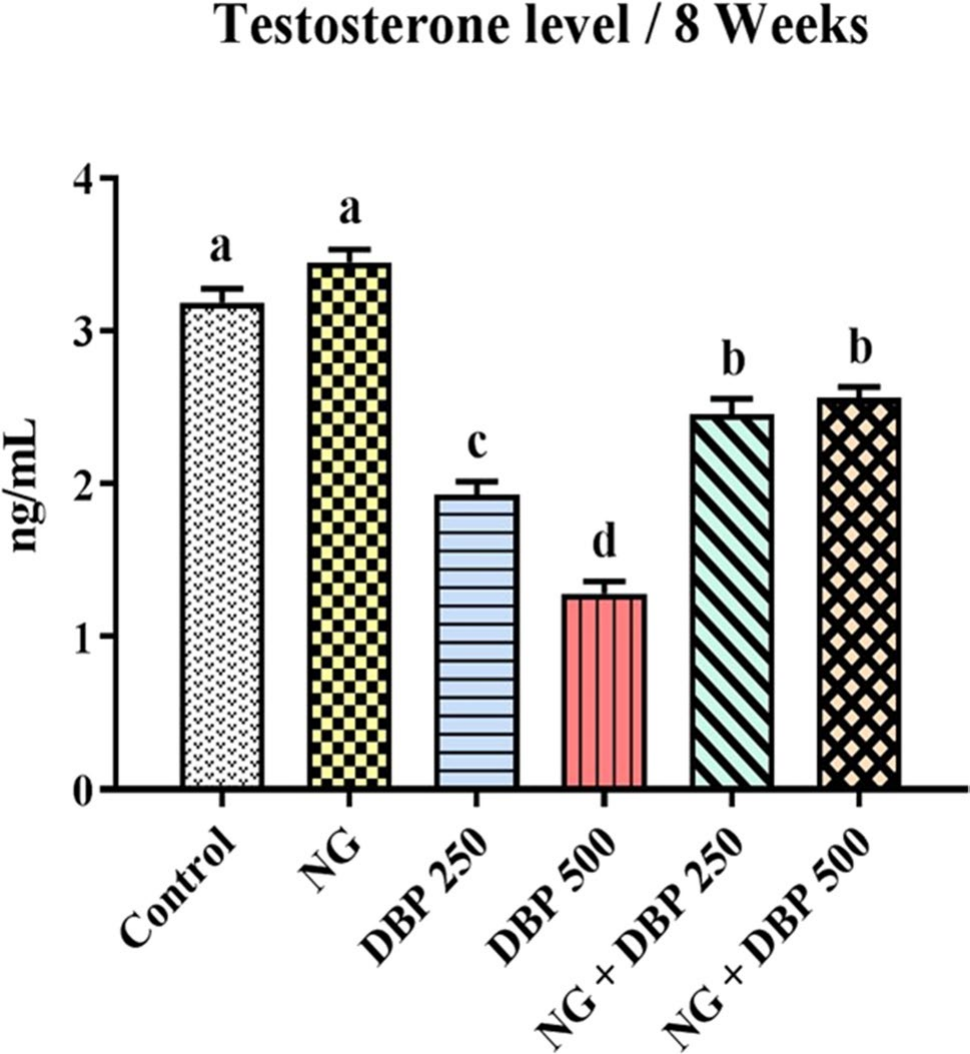
Effect of naringin and/or di-n-butylphthalate on serum testosterone level of the different treated groups. Values are expressed as mean ± SE (*n* = 7). Different superscripts (a, b, c, d) indicate significant differences at *p* < *0.05*. NG: naringin, DBP 250: di-n-butylphthalate (250 mg/kg), DBP 500: di-n-butylphthalate (500 mg/kg)

As displayed in Fig. 1, no significant changes were recorded in the serum testosterone level between control and NG-treated groups at *p* < *0.05*. Conversely, rats orally intoxicated with DBP at dose levels of 250 or 500 mg/kg induced a dose-dependent decrease in the serum level of testosterone, compared to control group. Oral administration of NG, one hour before DBP 250 or DBP 500 intoxication, significantly increased the serum testosterone level, compared to DBP 250 or DBP 500 groups, respectively, while still significantly different from the control value.

### Naringin improved the di-n-butyl phthalate-induced alterations in sperm quality

The alterations in sperm quality, including sperm count, viability and motility are recorded in Table 1. Regarding the control values, rats administrated NG did not show any significant changes in all sperm parameters except significant increase in the mean percentage of sperm rapid motility and significant decrease of the mean percentage of immotile sperm were observed at *p* < *0.05*. Concerning the normal control values, oral administration of DBP at dose levels of 250 or 500 mg/kg, induced marked alterations in the sperm quality evidenced by significant dose-dependent decreases in the sperm count and the mean percentages of sperm viability, rapid, slow and total motility with a significant increase in the mean percentage of immotile sperm. Interestingly, co-administration of NG with DBP 250 or DBP 500 significantly improved the sperm quality and restored the normal control values of sperm count, viability and motility, compared to DBP 250 or DBP 500 groups, respectively.

**Table 1:** Effect of naringin and/or di-n-butylphthalate on sperm count, viability and motility of the different treated groups

	Control vehicle	NG	DBP 250	DBP 500	NG + DBP 250	NG + DBP 500
Count (× 10^6^)	44.28 ± 1.30 ^a^	49.28 ± 2.50 ^ab^	32.00 ± 0.95 ^c^	24.71 ± 1.37 ^d^	42.14 ± 1.84 ^a^	40.71 ± 2.02 ^a^
Viability (%)	81.62 ± 1.02 ^a^	84.37 ± 1.20 ^a^	60.00 ± 1.17 ^b^	50.00 ± 2.04 ^c^	77.50 ± 1.54 ^a^	75.25 ± 1.55 ^a^
Rapid motility (%) (Grade a)	17.14 ± 1.48 ^b^	24.28 ± 1.30 ^a^	7.86 ± 1.84 ^c^	3.33 ± 0.97 ^d^	19.37 ± 2.70 ^b^	20.00 ± 1.19 ^b^
Slow motility (%) (Grade b)	29.28 ± 2.02 ^a^	29.27 ± 2.01 ^a^	15.71 ± 3.17 ^b^	10.83 ± 0.77 ^c^	25.00 ± 1.44 ^a^	23.33 ± 1.54 ^a^
Non-progressive motility (%) (Grade c)	33.33 ± 2.58 ^a^	30.00 ± 1.69 ^a^	34.00 ± 1.85 ^a^	28.00 ± 1.03 ^a^	31.00 ± 2.30 ^a^	32.00 ± 1.03 ^a^
Total motility (%) (Grade a, b, c)	79.75 ± 6.08 ^a^	83.55 ± 5.00 ^a^	57.57 ± 6.86 ^b^	41.71 ± 2.77 ^c^	75.37 ± 6.44 ^a^	75.33 ± 3.76 ^a^
Immotile sperm (%) (Grade d)	20.00 ± 0.77 ^d^	17.33 ± 2.22 ^c^	42.50 ± 4.89 ^b^	58.29 ± 1.69 ^a^	24.28 ± 1.78 ^d^	25.08 ± 2.23 ^d^

Values are expressed as mean ± SE, (*n* = 7). Different superscripts (a, b, c, d) indicate significant differences at *p* < *0.05*. NG: naringin, DBP 250: di-n-butylphthalate (250 mg/kg), DBP 500: di-n-butylphthalate (500 mg/kg)

### Naringin ameliorated the di-n-butyl phthalate-induced alterations in testicular oxidant/antioxidant status

**Fig. 2:**
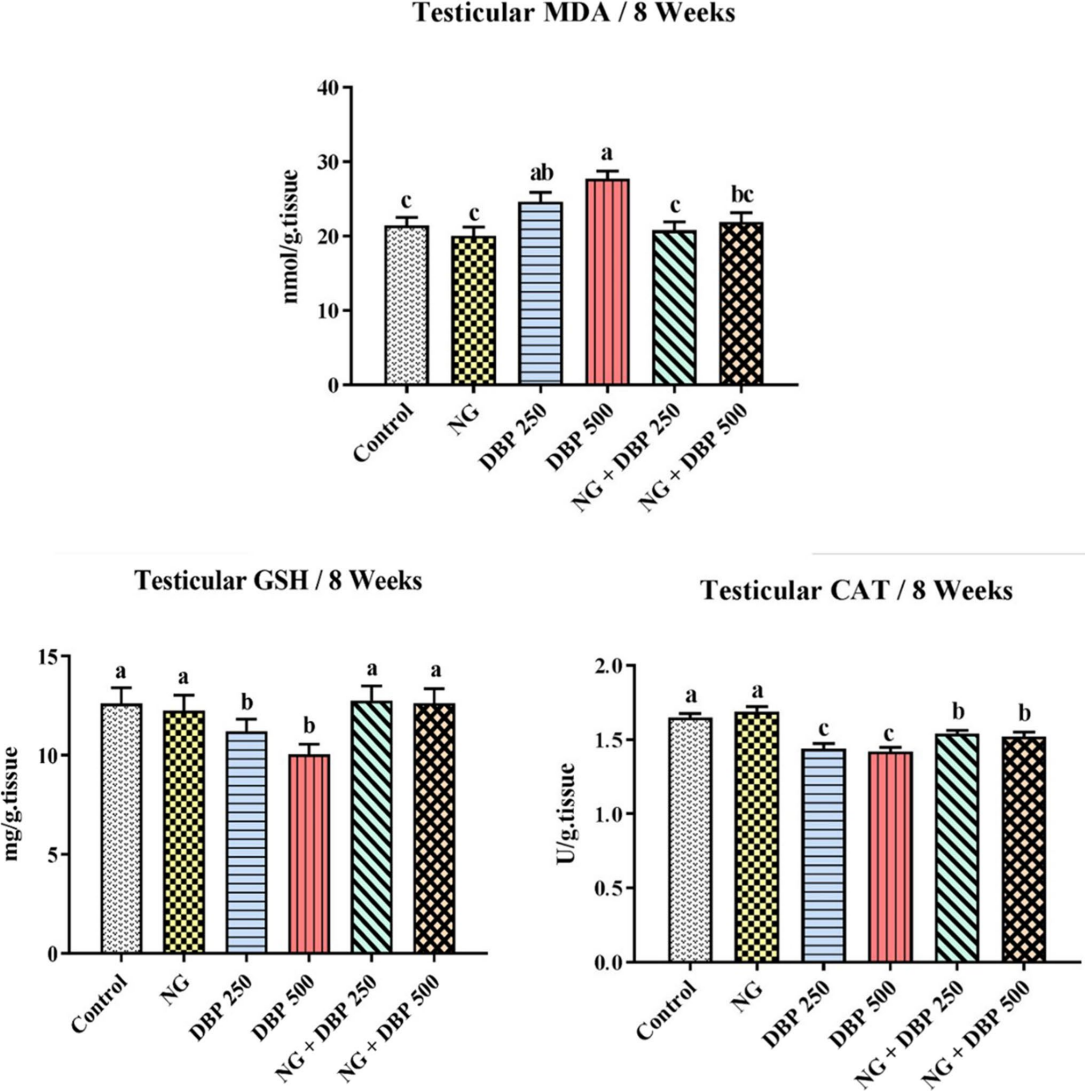
Effect of naringin and/or di-n-butylphthalate on testicular oxidant/antioxidant status of the different treated groups. Values are expressed as mean ± SE (*n* = 7). Different superscripts (a, b, c) indicate significant differences at *p* < *0.05*. NG: naringin, DBP 250: di-n-butylphthalate (250 mg/kg), DBP 500: di-n-butylphthalate (500 mg/kg)

Referring to the changes of the testicular oxidant/antioxidant status, oral administration of NG showed no significant *(P* < *0.05*) variations in the mean values of MDA level, GSH content or CAT activity in testicular tissue homogenates, when compared with the control values. In contrast, rats intoxicated with DBP 250 or DPB 500 exhibited significant dose-dependent increase in the mean values of MDA content along with significant reduction in the mean values of GSH content and CAT activity, compared to the corresponding values of the control group. On the other hand, the concurrent administration of NG with either DBP 250 or DPB 500, significantly ameliorated the DPB-induced alterations in the testicular oxidant/antioxidant status and normalized the values of MDA level and GSH content (Fig. 2).

### Naringin improved the di-n-butyl phthalate-induced alterations in testicular histoarchitectures

**Fig. 3:**
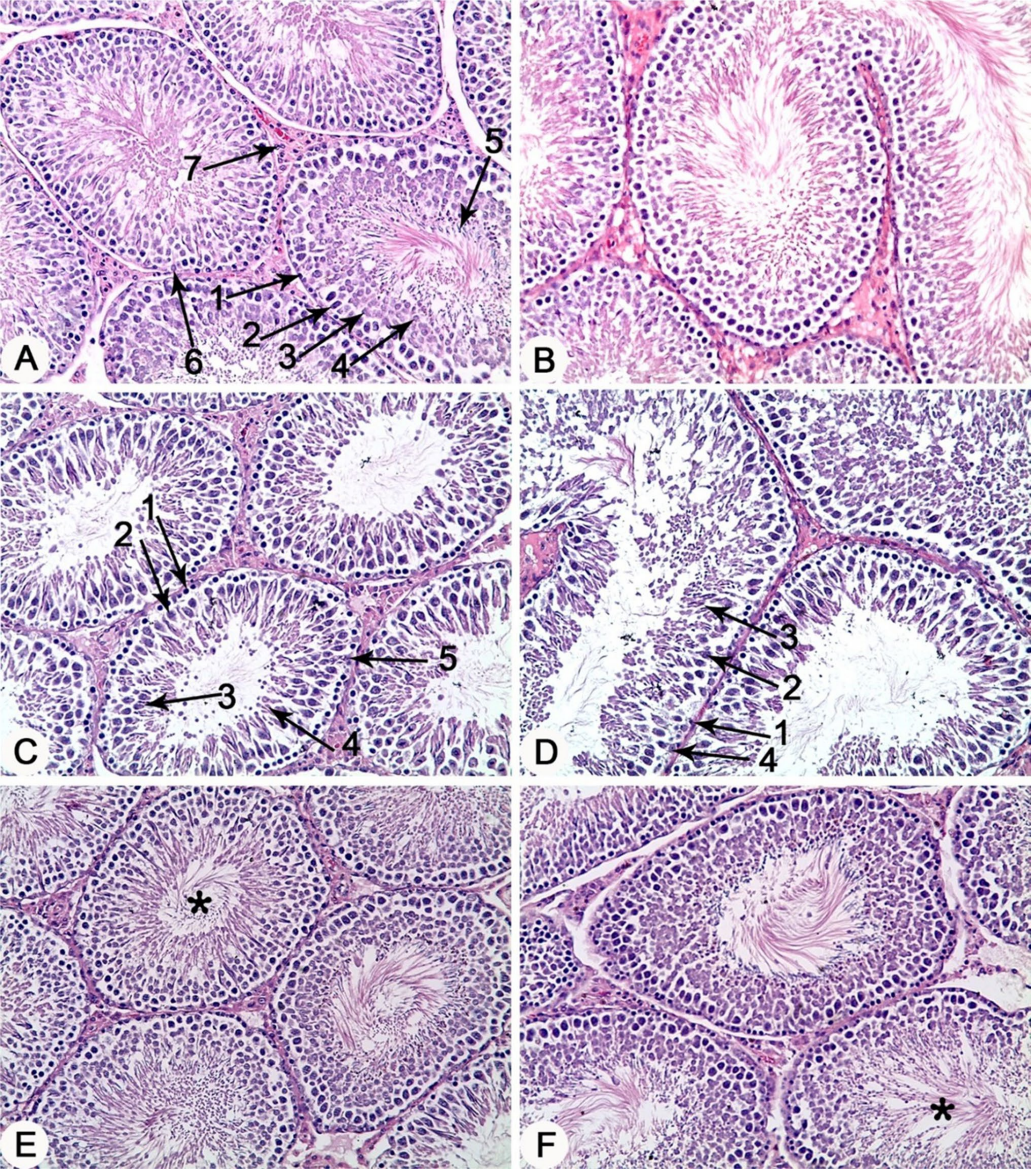
Testes, Rat. **A** & **B**) Control vehicle and Naringin-treated groups, respectively, showing normal histological architectures. 1, spermatogonia; 2, primary spermatocyte; 3, secondary spermatocyte; 4, spermatid; 5, spermatozoa; 6, Sertoli cells; 7, Leydig cells. **C)** DBP 250-treated group showing absence of the spermatozoa from the lumen of seminiferous tubules and degenerative changes in the secondary spermatocyte and spermatid cell layers. 1, spermatogonia; 2, primary spermatocyte; 3, secondary spermatocyte; 4, spermatid; 5, Sertoli cells. **D)** DBP 500-treated group showing complete absence of the spermatozoa from the lumen of seminiferous tubules and degeneration in all cell types of the seminiferous tubule’s epithelium. 1, spermatogonia; 2, primary spermatocyte; 3, secondary spermatocyte; 4, Sertoli cells. **E)** NG + DBP 250- and **F)** NG + DBP 500- treated groups showing mild decrease of spermatozoa (asterisk). H&E stain, X 10

Normal testicular tissues were observed in control and NG- treated groups (Fig. 3A & B). Conversely, absence of the spermatozoa from the lumen of the tubules, along with degenerative changes in the secondary spermatocyte and spermatid cell layers were observed in the seminiferous tubules of DBP 250- intoxicated rats (Fig. 3C). While, seminiferous tubules of DBP 500-intoxicated rats showed complete absence of the spermatozoa from the lumen of seminiferous tubules and degeneration of all cell types of the seminiferous tubules epithelium (Fig. 3D). On the other hand, co-administration of NG with either DBP 250 or DBP-500 showed only a mild decrease of spermatozoa in the lumen of the seminiferous tubules (Fig. 3E and F).

### Naringin ameliorates the di-n-butyl phthalate-induced alterations in the morphometry of seminiferous tubule’s epithelium

**Fig. 4:**
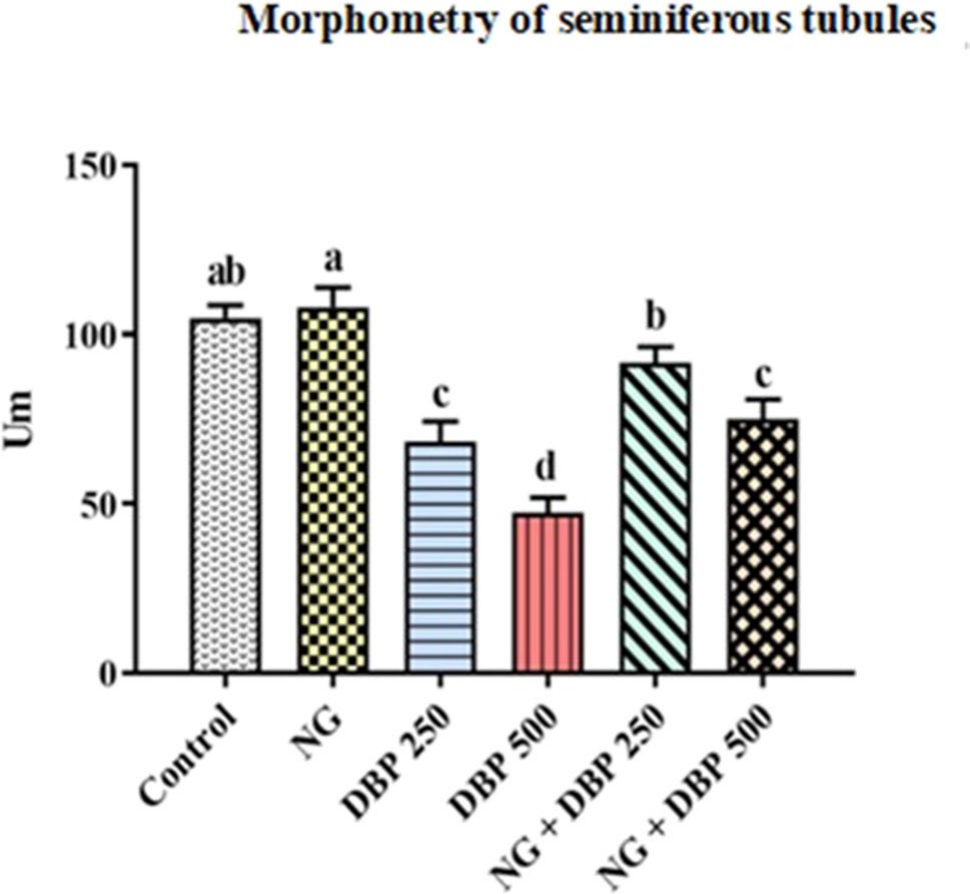
Morphometry of seminiferous tubules epithelium of the different treated groups, Um. Values are expressed as mean ± SE (*n* = 7). Different superscripts (a, b, c, d) indicate significant differences at *p* < 0.05. NG: naringin, DBP 250: di-n-butylphthalate (250 mg/kg), DBP 500: di-n-butylphthalate (500 mg/kg)

Regarding data presented in Fig. 4, no significant variations in the thickness of seminiferous tubule’s epithelium were observed between control and NG- treated groups. Nevertheless, rats intoxicated with DBP 250 or DBP 500 showed significant dose-dependent decreases in the thickness of seminiferous tubules epithelium, compared to control group. However, co-administration of NG either with DBP 250 or DBP 500 significantly increased the thickness of seminiferous tubules epithelium in comparison with those of rats intoxicated with DBP 250 or DBP 500 alone, respectively (Fig. 4).

## Discussion

Male reproductive disorders are a common public health problem and a considerable health challenge that has gained global attention over the decades (Akinola et al. 2010). Exposure to environmental pollutants, mainly the EDCs is among the main causes of male reproductive disorders all over the world **(**Green et al. 2021). Hence, there are growing concerns for the development of natural remedies to alleviate such disorders. Therefore, this study aimed to investigate the possible protective effect of NG against DBP-induced testicular damage and impairment of spermatogenesis in rats.

The current study clearly demonstrated the male reproductive toxic effect of DBP, evidenced by its significant adverse effects on serum testosterone level, sperm quality and testicular structure, which was further explained by the induction of oxidative stress. Our results revealed that oral administration of rats with DBP at dose levels of 250 or 500 mg/kg for 2 months significantly decreased, in a dose-dependent manner, the serum level of testosterone concomitantly with significant decrease in the sperm motility, sperm count and sperm viability. Similarly, Aly et al. (2016) reported a significant dose-dependent decrease in serum testosterone level with impairment of sperm quality in adult rats treated orally with DBP at doses of 200, 400, or 600 mg/kg/day for 15 consecutive days. Moreover, Nelli and Pamanji (2017) recoded that intraperitoneal injection of DBP (100 and 500 mg/kg), markedly decreased serum testosterone level with significant reductions of sperm count, motility and viability in adult rats. Testosterone, the main steroid male sex-hormone, is secreted by Leydig cells of the testes under the control of complex neuroendocrine interactions and is responsible for male sexual activity (El-Kashoury et al. 2010). The present findings revealed a pronounced decrease in serum testosterone level following exposure of male rats to DBP perhaps due to the activation of estrogen receptors (Alam et al. 2010), direct adverse effect of the DBP on testicular Leydig cells (Bielanowicz et al. 2016), inhibition of 3β-HSD and 17β-HSD enzyme, which are essential for steroidogenesis and testosterone biosynthesis (Nelli and Pamanji 2017), or due to the induction of oxidative stress (Aly et al. 2016). Evaluation of the sperm quality indices, including sperm motility, sperm count and sperm viability is valuable indicators of the testicular function and the male reproductive performance (Jenardhanan et al. 2016). The testicular function is controlled by the gonadotrophic hormones, luteinizing hormone (LH) and follicle-stimulating hormone (FSH) (Oduwole et al. 2018). LH stimulates Leydig cells for testosterone production while FSH, in concert with testosterone, regulates spermatogenesis via Sertoli cell function (Oduwole et al. 2018). Previous studies reported significant reductions of LH and FSH levels following exposure to DBP (Aly et al. 2016; Nelli and Pamanji 2017), resulting in alterations in the function of Sertoli and Leydig cells (Bielanowicz et al. 2016). Herein, the observed reduction of sperm motility, count and viability could be explained by the recorded reduction in testosterone level, suggesting alterations in pituitary gonadotropins that lead to a reduction in serum testosterone level, which consequently contributes to the impairment of spermatogenesis (Nelli and Pamanji 2017). Additionally, Aly et al. (2016) attributed the reduction in sperm motility and the inhibition of spermatogenesis in DBP-exposed rats to the impairment of Sertoli cells functions, which cause a decrease in the activity of testicular lactate dehydrogenase (LDH), a testicular germ cell enzyme, which is essential for the metabolic activity and motility of mature sperms (Kumar et al. 2013).

Further, these results were well substantiated by the histopathological alterations observed in the testicular tissue of rats exposed to both doses of DBP. Degenerative changes in the secondary spermatocyte and spermatid cell layers along with absence of the spermatozoa from the lumen of the seminiferous tubules were recorded in testicular tissue of rats orally intoxicated with DBP at a dose 250 mg/kg. However, the seminiferous tubules of rats intoxicated with DBP at a dose of 500 mg/kg showed degenerative changes of all cell layers as well as absence of the spermatozoa from the lumen. In line with these findings, Zhou et al. (2010) observed pathological changes included seminiferous tubule atrophy, shedding of the seminiferous epithelial cells, decrease of spermatogenic cells, and oligozoospermic lumen following oral exposure of rats to 500 mg/kg DBP for 2 weeks. Similarly, dose dependent degenerative changes in the seminiferous tubules with absence of spermatogenic cells and sperms were recorded following DBP exposure (Aly et al. 2016). In addition to the degenerative changes of seminiferous tubules observed in the current study, rats intoxicated with DBP 250 or DPB 500 showed significant dose-dependent decreases in the thickness of seminiferous tubule’s epithelium, as recoded previously by Nelli and Pamanji (2017), who observed a dose-related decrease in the diameter and epithelial layer thickness of seminiferous tubules of DBP-intoxicated rats at 100 and 500 mg/kg.

Recently, abundant literatures reported oxidative stress among the mechanisms by which DBP- induced testicular damage and impairment of spermatogenesis (Aly et al. 2016; Nelli and Pamanji 2017). Our findings demonstrated a dose-dependent elevation of MDA level concurrently with significant reductions of GSH content and CAT activity in testicular tissue of rats administrated DBP at 250 mg/kg and 500 mg/kg. The accomplished results are closely resembled to those obtained by Zhou et al. (2010); Aly et al. (2016); Nelli and Pamanji (2017), who recorded that exposure to different dose levels of DBP induced oxidative testicular damage and impairment of spermatogenesis. The shift in balance between the production and scavenging of reactive oxygen species (ROS) is termed oxidative stress that contributes to many toxicological and pathological conditions (Birben et al. 2012). The high content of polyunsaturated fatty acids in plasma membrane of sperm promotes its susceptibility to oxidative damage and lipid peroxidation that closely correlated to the reductions in sperm motility, count and viability (Bansal and Bilaspuri 2011). However ROS has an important role in regulating the normal sperm function, excess production of ROS may induce oxidative stress and lipid peroxidation, leading to adverse effects on sperm quality and male fertility (Bansal and Bilaspuri 2011). Furthermore, ROS may induce apoptosis of sperms and germ cells, possibly by disturbing the inner and outer mitochondrial membranes and affecting the balance between pro- and anti-apoptotic proteins (Asadi et al. 2021). The obtained findings in this study suggested the implication of oxidative stress in DBP- induced testicular damage and impairment of spermatogenesis, mainly via the excessive ROS production and the suppression of the antioxidant enzymatic defenses, causing damage of sperm and spermatogenic cells, which in turn, cause reduction of testosterone level and reduction of sperm motility, count and viability.

Interestingly, the concomitant treatment with NG at a dose level of 80 mg/kg, one hour before DBP 250 or 500- intoxication, mitigated the dose-dependent reproductive dysfunctions induced by DBP, reflected by significant increases of serum testosterone level, sperm motility, count and viability along with a significant decrease in testicular MDA level concomitantly with significant increases in GSH content and CAT activity. This was accompanied by marked improvement of the testicular histoarchitecture and significant increase in the seminiferous tubules epithelial thickness.

In agreement with our findings, Akondi et al. (2011) proved that oral administration of rats with NG (5, 10 mg/kg) for 35 days mitigated, in a dose-dependent manner, the reproductive toxic effects of gentamycin, indicated by the increase of sperm vitality, sperm motility and sperm count percentages, reduction of testicular MDA level with increases of SOD and catalase activities as well as improvement of the testicular architecture. Meanwhile, administration of NG at dose levels of 40, 80, 160 mg/kg for 30 days improved the testicular function and structure in BPA-intoxicated rats, proved by the increased the epididymal sperm count, testicular enzymes, serum hormonal levels, antioxidant enzymes activities and improved the testicular morphology with increasing the diameter, and epithelial height of seminiferous tubules (Alboghobeish et al. 2019). Noteworthy, previous literatures proved also the protective effect of naringenin, the main metabolite of NG, against the impairment of the function and histological architecture of testes induced by hydrogen peroxide toxicity (Sahin et al. 2017) or by the mobile phone electromagnetic waves (Farag and Yousry 2018).

The recoded improvement in sperm motility, count and viability might be due to obvious protective effect of NG on the Leydig cells, which accompanied by releasing more testosterone, which is essential for normal spermatogenesis (Farag and Yousry 2018) and also could be due to the suppression of ROS-induced disturbances in sperm and germ cells (Alboghobeish et al. 2019). It is well-known that antioxidants protect DNA and other important molecules from oxidative damage, and can improve sperm quality, and consequently, increase male fertility (Bansal and Bilaspuri 2011). In this regard, the reproductive protective effect of NG recorded in the current study could be attributed mainly to its antioxidant activity that reflected by the recorded reduction of testicular MDA level and the increments of GSH content and CAT activity. This explanation was in line with previous reports, which proved the antioxidant activity of NG and its metabolite, naringenin (Ahmed et al. 2019; Alboghobeish et al. 2019) that may be related to their ability to reduce the production of ROS by coupling with Cu and Fe ions through the 5-hydroxy and 4-carbon groups in their C ring (Mostafa et al. 2016). Also, NG could eliminate the produced ROS by its free radical scavenging property (Cavia-Saiz et al. 2010). The NG has also been recorded to enhance the expression of several antioxidant-related genes and suppress the activity of ROS-forming enzymes such as NADPH oxidase (Ciz et al. 2012). Moreover, Ahmed et al. (2019) attributed the protective effect of both NG and naringenin to the improvement of antioxidant defense system and the suppression of inflammation and apoptosis pathways. All the above studies clearly hypothesized the mechanistic role of the antioxidant effect of NG in improving the testicular function and structure.

## Conclusion

The findings recorded herein proved that oxidative stress and the subsequent reduction in testosterone secretion are among the potential underlying mechanisms of DBP-induced testicular damage and impairment of spermatogenesis. Also, these findings indicated the protective effect of NG, suggesting the perspective of using NG as a natural protective and therapeutic agent for alleviating reproductive dysfunctions and improving the reproductive performance, mainly via its potent antioxidant activity.

## Data Availability

The authors confirm that all data supporting the finding of this study are available within the article and there are no other supplementary data.
